# An overview of agricultural neonicotinoid regulation in the EU, Canada, and the United States

**DOI:** 10.1002/ps.70126

**Published:** 2025-08-12

**Authors:** Katherine Dentzman, Derek Franklin, Edem Avemegah, Jessica R. Goldberger

**Affiliations:** ^1^ Department of Sociology and Criminal Justice Iowa State University Ames IA USA; ^2^ Department of Crop and Soil Sciences Washington State University Pullman WA USA

**Keywords:** agriculture, pollinators, restrictions, governance, neonicotinoids, regulation, review

## Abstract

Neonicotinoids are a widely used class of insecticides partly due to their low acute risk to non‐target organisms. However, increasing concerns over their long‐term chronic effects on pollinators and other species of concern have led to increased governmental regulations since the mid‐2010s, particularly in agricultural settings. These regulations have varied in terms of approach, geography, and timeline, starting with a ban implemented by the European Union (EU) and evolving into exemption‐based regulations across two Canadian provinces and five US states as of this writing. While the landscape of neonicotinoid regulation in agriculture is rapidly evolving, it is pertinent to review what contexts led to different regulatory approaches in each of these cases, what templates for regulation exist, and what the consequences of such approaches have been up to this point. This review aims to enhance understanding of the potential future of agricultural neonicotinoid regulation across time and space. © 2025 The Author(s). *Pest Management Science* published by John Wiley & Sons Ltd on behalf of Society of Chemical Industry.

## Graphical abstract

Grounded in broad environmental coalition and place‐based research, the European Union, Ontario and Québec in Canada, and several US states placed restrictions on agriculturally‐applied neonicotinoids. The exact regulations and how they were implemented are explored.

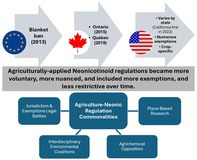

## 1 INTRODUCTION

Neonicotinoids—a widely used class of neurotoxic systemic insecticides designed to control piercing and sucking pests—are facing increasing scrutiny, particularly regarding their application in agricultural settings.^1^, ^2^, ^3^ Although neonicotinoids exhibit low acute toxicity to non‐target organisms, there is growing concern about their long‐term environmental effects and impacts on non‐target species.^4^, ^5^ These encompass concerns such as chronic sublethal exposure and its potential impacts on pollinator health, the health of aquatic insects and crustaceans, cumulative toxicity in birds and mammals, and potential human health effects.^1^, ^6^, ^7^, ^8^, ^9^, ^10^, ^11^, ^12^, ^13^ There is evidence that different common neonicotinoid derivative groups pose differing levels of threats, with nitroguanidine neonicotinoids (including clothianidin, dinotefuran, imidacloprid, and thiamethoxam, often applied as seed treatments) presenting greater risks to pollinators than the cyanoamidine group (including acetamiprid and thiacloprid, mainly used as foliar applications).^14^ The cyanoamidine group has demonstrated lower toxicity to bees, but has also been found to have high toxicity to aquatic insects.^14^ Limited research in certain areas, however—for example, nitroguanidine neonicotinoids are better studied than the cyanoamidine group—hinders a comprehensive understanding of the relationship between these ecological issues and neonicotinoids.^15^ However, limited research in certain areas hinders a comprehensive understanding of the relationship between these ecological issues and neonicotinoids.

Despite ongoing controversy over whether these risks are exaggerated, especially considering different neonicotinoid groups as well as the alternatives used when neonicotinoids are restricted, there has been a growing number of proposed and enacted regulatory schemes that limit, or in some cases ban, the use of certain neonicotinoids in both agricultural and ornamental applications.^16^, ^17^, ^18^, ^19^, ^20^ Indeed, neonicotinoids present a unique regulatory case due to their sometimes systemic nature, documented risk to pollinators, widespread agricultural application, and high levels of public and scientific scrutiny.^21^

The current state of international regulatory approaches for neonicotinoids in agriculture is a rapidly changing landscape that is challenging to monitor and categorize. However, reviewing current and proposed regulatory schemes at the state and federal levels is nonetheless a key resource for understanding the possibilities, consequences, and successes of different potential governance structures regulating neonicotinoid use. It is increasingly apparent that public concern regarding the plight of pollinators, as well as aquatic invertebrates, is growing, with public and private entities responding accordingly.^22^, ^23^ This paper reviews the status of neonicotinoid regulation using the cases of the European Union, Canada, and the United States (US). Due to the promulgation of province‐ and state‐level regulation in Canada and the US, individual province and state programs targeting the regulation of neonicotinoids in agricultural contexts are also discussed.

## 2 REGULATORY CASES

### 2.1 The case of the European Union

Authorization for the use of the neonicotinoid imidacloprid was obtained in some EU countries as early as 1991; other forms of neonicotinoids, such as thiamethoxam, were authorized throughout the late 1990s and early 2000s using European Plant Protection Organization (EPPO) testing protocols.^24^ In 2009, multiple national‐level research institutes reported to the European Food Safety Authority (EFSA) that there was a need to improve the knowledge and understanding of factors affecting bee health.^21^ Four years later, the EFSA published a report based on a series of experiments demonstrating systemic links between neonicotinoids and bee health. Several other research efforts involving collaborations among ecologists, entomologists, and biochemists had been published in the intervening years with similar conclusions.^21^ These researchers widely shared their findings with bureaucratic working groups, NGOs, and agencies such as the European Environment Agency. At the same time, public interest in bees was growing, particularly through journalistic pieces chronicling the organized work of beekeepers dedicated to raising awareness about the impacts of insecticides on bee health.^21^ These beekeepers and their associations engaged with public researchers, alternative agricultural public interest groups, and specialized NGOs to gain the ear of members of the European Parliament.^21^

The result of these cumulative efforts was EU Regulation 485/2013, which placed a moratorium on the use of three specific neonicotinoids (imidacloprid and clothianidin, manufactured by Bayer, and thiamethoxam, manufactured by Syngenta) in bee‐attractive open‐crop fields.^17^, ^21^, ^24^ Specifically, the use of nitroguanidine neonicotinoids was prohibited in corn/maize, canola, and sunflower crops.^15^ Controversy arose, particularly because the decision was contested by agrichemical industries. Subsequent field studies were conducted, with agrichemical industries contending that the results demonstrated the in‐field safety of neonicotinoids.^24^ However, this interpretation was roundly criticized by non‐industry scientists and citizens, and in 2018 the EFSA conducted a reassessment of existing evidence on neonicotinoids and bee health. Based on this reassessment, EU Member States approved a full ban of the imidacloprid, clothianidin, and thiamethoxam in outdoor applications.^24^ Cyanoamidine neonicotinoids have not been restricted in the EU, and acetamiprid was approved through February 28, 2033.^15^, ^25^, ^26^ Two additional neonicotinoid derivative groups, sulfoxaflor and flupyradifurone, are currently up for renewal of their approval as of 2025.^26^

Exceptions to the EU ban include allowance for use in greenhouses, the treatment of some crops post‐flowering, and applications in winter cereals and sugar beets; eventually, these exceptions were reduced to indoor use only.^17^ There have been numerous ongoing discussions regarding these exceptions; for instance, in December 2020, the EU Court of Justice ruled in favor of permitting the use of imidacloprid and thiamethoxam in sugar beets due to massive infestations of disease‐carrying aphids. As a result, 236 emergency pesticide exemptions were made between 2019 and 2023, with 47.5% of granted exemptions being for neonicotinoids.^27^, ^28^ Following appeals from farmers, beekeepers, and environmental organizations, the decision for pesticide exemptions was reversed in 2023, and it was also determined that individual European countries could not legally grant exceptions for the use of pesticides that the EU has banned, aligning with recommendations by Epstein *et al*. to stringently limit emergency exemptions.^29^, ^30^

One factor in the decision to limit exemptions was the detection of neonicotinoids at levels above environmental quality standards deemed safe for aquatic wildlife in 10 English rivers, indicating that ban compliance may be spotty; long‐term persistence of neonicotinoids in soil and water may also have contributed.^31^ Additionally, research has found high levels of soil and canola nectar contamination, particularly with imidacloprid, even after the 2013 and 2018 bans took place, suggesting that partial bans (*i.e*. those not extending to non‐pollinated crops and indoor use, or granting too many emergency exemptions) may be insufficient to adequately control risks.^32^ France proactively accounted for these risks by additionally banning neonicotinoids in public spaces, greenhouses, and non‐agricultural activities and is investing significant time and funding to find appropriate alternatives.^33^

The United Kingdom (UK) is no longer part of the European Union and has thus been able to grant neonicotinoid exemptions since 2021.^34^ However, there have still been several rounds of exemptions and recensions of these exemptions.^35^ For example, in 2024 neonicotinoids could be applied to sugar beets when there was a scientifically verified level of threat posed by yellows virus, defined as 65% infection across the national sugar beet crop.^35^ However, the exemption was not renewed for 2025 due to uncertainty over the weight of risks *versus* benefits.^36^ Two neonicotinoids currently allowed are up for renewal evaluation in 2025 and the EU has also announced intentions to ban, by 2026, the import of products containing any traces of thiamethoxam and clothianidin^26^, ^37^.

As one of the longest and most comprehensive neonicotinoid bans in the world, the EU case has attracted some emerging studies on agronomic and economic impacts. For instance, the ban has been found to result in costs of $977 million for EU canola farmers between 2013 and 2017.^38^ This statistic, from a study commissioned by Bayer and Syngenta, also catalogs other impacts of the neonicotinoid ban.^16^ These include a yield loss ranging from <1% to 20% with an average of 4%, or about 912 000 tons of canola; an average of 6.3% harvest quality loss; and additional foliar applications of other insecticides such as pyrethroids.^39^, ^40^ Many growers have also chosen to move to other crops, which has resulted in increased imports of canola—imports grew from 63 000 tons in 2016 to 811 000 tons in 2022.^38^ Overall, these losses have been estimated to be in the range of €510 million per year, with the majority coming from market revenue losses.^40^ Similarly, it is estimated that losses related to banning neonicotinoids for sugar beets could result in 8.8% of sugar beet crops due to virus yellows over the next 75 years.^41^ There are exceptions, however—banning neonicotinoids in winter and spring canola in Sweden did not have any major discernable impacts on total cropping area or crop yields per hectare, potentially due to a harsher winter climate limiting pest overwintering as well as limited pyrethroid resistance in the country.^42^

Beyond yield and economic impacts, several assessments have determined that banning neonicotinoids in the EU can result in growers switching to other pesticides, such as pyrethroids—sometimes at higher rates—which also have major toxicology risks.^43^, ^44^, ^45^, ^46^ A pesticide risk assessment mapping the pesticide load of 59 pesticides on 28 crops and pastures in the EU found that recent bans on 14 pesticides could significantly reduce pesticide loads by 94% if the banned pesticides were replaced with non‐pesticide‐based control practices. However, the bans also have the potential to *increase* pesticide loads or merely shift risks as other pesticides take the place of neonicotinoids.^47^ Supporting this finding, Andert and Ziesemer's study of pesticide application in canola in Northeastern Germany pre‐ and post‐ban found that insecticide applications—usually of chlorantraniliprole—increased by an average of 22.5% from 2012 to 2019.^48^ Likewise, in a case study of seven EU countries, Kathage *et al*. found that farmers generally relied on alternative seed treatments or more frequent soil and foliar treatments to control early‐season pests in sugar beets.^43^ However, despite such increases in pesticide application, pollinators may still benefit; Germany's Total Applied Toxicity risk for pollinators dropped substantially after the ban, although it should also be noted that France's did not, presumably as the result of a turn towards pyrethroids.^49^

### 2.2 The case of Canada

Neonicotinoids were first used in Canada to control the Colorado potato beetle and flea beetle in potatoes and canola in the 1990s.^33^ This use was swiftly followed by a growing body of work showing negative impacts on bees.^50^ However, agrichemical companies have argued that there is too much scientific uncertainty to be conclusive.^50^ Nonetheless, the province of Ontario followed the example of the EU in that Ontario attempted to impose a blanket ban on nitroguanidine neonicotinoids, but some restrictions were later rescinded.^50^ Ultimately, Ontario instituted a partial ban based on the precautionary principle, *i.e*., that a given course of action or technology must be proven safe before it can be used, to reduce agricultural use of neonicotinoids by 80%.^50^

A key factor in Ontario's ban was the Ontario Bee Working Group, established by the provincial government in 2013. The Ontario Bee Working Group included participants from the Ministry of Agriculture and Rural Affairs, the Ministry of the Environment, the University of Guelph, the Ontario Beekeepers’ Association, the Ontario Federation of Agriculture, Syngenta, Bayer Crop Science Canada, Crop Life Canada, the Association of Equipment Manufacturers, and the Canadian Seed Association.^50^ There was also significant support from other stakeholders, including an advertising campaign supporting a neonicotinoid ban from the Canadian Association of Physicians for the Environment and Registered Nurses of Ontario.^51^ Public support was also high; an Ontario Omnibus Survey Report from 2014 indicated that 76% of Ontario residents expressed concern about the future of bees, that the rate of concern was higher in agricultural areas, and that 77% supported the Ontario Government's proposal to reduce neonicotinoid use by 80% from 2015 to 2017.^51^

In 2014, the Ontario Bee Working Group made 13 recommendations to the provincial government regarding the use of neonicotinoids, including the recommendation for a temporary ban for the 2014 growing season. A period of public consultation followed, during which beekeepers, farmers, and concerned citizens provided feedback on the proposed ban. Ontario's ban went into effect on July 1, 2015, and despite being contested by the Grain Farmers of Ontario, the ban stayed in place.^51^ The Ontario Bee Working Group ultimately settled on a suite of primarily voluntary protective measures, potentially due to the involvement of multiple agrichemical companies and significant pesticide lobby marketing.^50^, ^51^ It was decided that farmers wishing to buy and use neonicotinoid‐treated seeds must complete a one‐time free Integrated Pest Management (IPM) training, complete a pest risk assessment report, and sign an IPM Written Declaration Form stating that they have considered IPM principles to decrease the risk of early season insect damage.^52^ The pest risk assessment report includes three risk assessment methods: soil pest scouting, crop damage assessment, and pest risk criteria. Other criteria for neonicotinoid‐treated see waiver approval were listed as: an average of two or more grubs or one wireworm in the soil at a farm property, at least a 15% stand loss in corn or a 30% stand loss in soybean as the result of pest, and/or the farm fits specific pest risk criteria including soil type, cover crop usage, conservation tillage methods, and a record of past pest infestations. If a farmer's application to use neonicotinoid‐treated seeds is approved, they are only allowed to plant those seeds in the fields identified in their pest risk assessment report and maintain records of when the seeds are planted for a minimum of 2 years.^52^

Québec has also moved to independently regulate nitroguanidine neonicotinoid use, largely as a result of Health Canada's 2012/2013 report on the negative impacts of neonicotinoids on bee health combined with a series of province‐level water contamination studies.^53^ In 2019, the province prohibited the use of neonicotinoid‐treated seeds for corn and soybean unless growers obtained a verification of need from an independent agronomist.^53^, ^54^, ^55^ This move was part of a larger Québec Pesticide Strategy and requires (1) the approval of an agronomist certified through the Ordre des Agronomes du Québec before neonicotinoids can be used in‐field and (2) a record of all pesticides applied. Additionally, seeds coated in neonicotinoids are now treated to the same regulations as other pesticides and Québec has prohibited residential and commercial use of neonicotinoids on lawns.^54^ The province allocated CAD $14 million over 5 years to assist farmers in adapting to these new regulations, now offers regular training sessions for agronomists and farmers, and has established a monitoring committee to oversee progress.^53^, ^54^, ^55^

Québec may be especially well‐adapted to ban neonicotinoids, as research has found that prophylactic neonicotinoid treatment in corn and soybean showed no significant differences in plant stand or yield compared to no treatment—potentially because relevant pest rates are particularly low in Québec's climate.^56^, ^57^ Perhaps because of this, adaptation to the neonicotinoid restrictions has been broadly accepted.^54^ Neonicotinoids were estimated to account for less than 0.5% of all corn and soybean seed planted in Québec in 2019; they have been replaced primarily by diamides (about 60% of corn fields in 2021) and completely untreated seed (about 20–30% in 2023).^53^, ^58^ A report by the Québec government found a drop in detected neonicotinoids in surface waters from 2018 to 2020 as a result of these changes.^59^ However, there is concern that diamides are as toxic as neonicotinoids and maybe more so.^59^ In response, Québec also plans to subject all insecticide seed coatings—not just neonicotinoids—in corn, soybean, canola, wheat, and barley to regulations, including agronomic justification and prescription by a trained agronomist, to go into effect in 2025.^53^

Beyond the provinces of Ontario and Québec, the Canadian federal government, after receiving large numbers of bee incident reports in 2012, considered restricting imidacloprid, clothianidin, and thiamethoxam use.^50^, ^60^, ^61^ After reviewing the science, including studies specific to Canada, Health Canada's Pest Management Regulatory Agency (PMRA), in 2018, proposed a phaseout of all agricultural, ornamental, and greenhouse use of neonicotinoids over the next 3–5 years.^60^ However, this proposal was heavily altered by the PMRA in 2021. The PMRA concluded that the neonicotinoids in question were largely acceptable, and only minor mitigations were required, such as restricting their application to seed treatments.^60^, ^61^, ^62^ This decision came after years of studying water monitoring data and fielding over 46 000 comments, with Health Canada concluding that most uses of imidacloprid met current human and environmental health and safety standards.^63^ Canada's PMRA additionally determined that they would not pursue a ban on clothianidin and thiamethoxam, but ‘Re‐evaluations of the health, environmental and value assessments of clothianidin and thiamethoxam are currently underway.’^61^

Investigative reporting by *Canada's National Observer* alleges that the reversal largely resulted from agrichemical company interference and collusion, including providing the federal government with private datasets to justify the continued use of neonicotinoids.^50^, ^60^, ^64^ Ultimately, the result was a compromise: PMRA proposed multiple risk mitigation measures for imidacloprid, including cancellation for some specific uses such as in‐furrow application on brassica, leafy, and root tuber vegetables, including potatoes. Additionally, there are new maximum application rates for specific seed treatments.^63^ An ongoing debate persists regarding the necessity of further restricting the use of neonicotinoids in Canadian agricultural production, and a reevaluation of these decisions is planned for publication in the second quarter of the 2025–2026 fiscal year.^61^, ^64^

### 2.3 The case of the United States

The US has generally been slower to implement restrictions on neonicotinoids than the EU or Canada. However, specific states have followed the lead of those countries by passing regulations at the state level. In 2008, the US Environmental Protection Agency's (EPA's) Environmental Fate and Effects Division, having been pushed on the issue by The Pesticide Action Network, acknowledged both nitroguanidine and cyanoamidine neonicotinoids’ potential risk to non‐target species and endangered aquatic vertebrates.^33^ The EPA's acknowledgment of ecological risks was followed by a 2014 Obama Administration memorandum creating a Pollinator Task Force, which produced a National Strategy to Promote the Health of Honeybees and Other Pollinators.^33^, ^65^ The presidential memorandum specifically charged the Pollinator Task Force with assessing the impact of neonicotinoids on the health of bees and other pollinators and suggesting specific actions to mitigate identified risks.^65^

Throughout 2014 and 2015, the EPA conducted a literature and data review exercise focused on determining the impacts of neonicotinoids on bees and other pollinators, pausing approval of applications for new uses of nitroguanidine neonicotinoids in the interim.^65^ Economic analyses conducted by the EPA also investigated the potential impacts of banning the use of neonicotinoids in soybeans, with time for public comments. Ultimately, the EPA proposed prohibiting the foliar application of acutely toxic products, including nitroguanidine neonicotinoids, during bloom for locations with bees on‐site unless the application is made in accordance with a government‐declared public health response.^65^ Beyond this national‐level proposal, the EPA recommended that more specific pollinator protection plans be developed by individual states and tribal agencies, with consultation from the EPA. As of 2023, at least 20 states had developed such plans or passed legislation protecting pollinator habitats.^66^ Other EPA measures included an expedited review of new products to control Varroa mites, improved guidance on reducing exposure during planting, and bee incident reporting.^65^

Some action has been taken since the National Strategy to Promote the Health of Honeybees and Other Pollinators was published in 2015. Notably, the Center for Food Safety brought a lawsuit that resulted in the accused registrants voluntarily petitioning the EPA to cancel the registrations for 12 pesticides containing the neonicotinoids clothianidin and thiamethoxam.^50^, ^67^ The EPA continues to review neonicotinoid pesticides to update their risk assessments and pursue risk mitigation as appropriate.^68^ In 2020, they released a list of proposed interim decisions for neonicotinoid use while their risk assessment review continues. This proposal included management measures to reduce risks, requiring additional personal protective equipment for applicators, restricting when pesticides can be applied to blooming crops, adding language to labels advising homeowners not to use neonicotinoids, and canceling spray uses of imidacloprid on residential turf.^68^ A registration review and amended proposed interim decision on the use of imidacloprid, clothianidin, thiamethoxam, dinotefuran, and acetamiprid are expected in 2025.^69^

There has also been increasing attention to pollinator health issues from private US corporations. A prominent example is Walmart, which developed a corporate pollinator health policy requiring its global fresh produce and floral suppliers to adopt IPM practices and be verified by a third‐party certifier by 2025.^70^, ^71^ The policy also encourages but does not currently require suppliers to track, report, and discontinue the use of neonicotinoids.^68^ Walmart has committed to tracking this use in its supply chain to boost transparency and assess annual progress.^70^ Specific to the IPM criteria, verification can be obtained from any third‐party certifier recognized by the IPM Institute of North America.^72^ Walmart's new requirements are representative of an increasing number of high‐profile voluntary efforts by corporate entities to regulate neonicotinoids.^73^, ^74^ However, there is some concern that private regulation may be ineffective given their profit orientations and limited formal power, along with the potential to take away public attention and decrease support for public regulation.^74^

Meanwhile, some individual US states have taken action related to neonicotinoid regulation. This paper briefly explores the evolution of these regulations, noting that the landscape is swiftly changing. Additionally, only regulations applying to agricultural uses are covered. Other states have enacted regulations on non‐agricultural uses, including Colorado, Maine, Maryland, Nevada, New Jersey, and Washington.^75^, ^76^, ^77^, ^78^, ^79^, ^80^

#### 2.3.1 California

In California, the Department of Pesticide Regulation (DPR) first began receiving adverse effects disclosures of neonicotinoids on pollinators in 2008; in 2009, four nitroguanidine neonicotinoids were subjected to reevaluation to determine any limitations on use for agricultural food and feed commodities.^81^ Proposed regulations resulting from this reevaluation went into effect on January 1, 2024, limiting soil and foliar—but not seed‐treated—nitroguanidine neonicotinoids in the production of agricultural commodities in 16 identified groups including fruits, vegetables, and cereal crops.^81^

Specific guidelines are provided for Category 1 crops that are typically harvested before bloom (no restrictions), Category 2 crops that are usually harvested after bloom (use prohibited during bloom), and Category 3 crops that the DPR was unable to determine whether applications are low risk to pollinators (use prohibited during bloom). For categories 2 and 3, regulations also include use restrictions disallowing more than one type of neonicotinoid active ingredient, more than one application method, and no application if managed pollinators will be used with the crop.^82^ These regulations do *not* apply to non‐agricultural and non‐production contexts (*e.g*., parks and cemeteries), nursery stock, cyanoamidine group neonicotinoids, or seed treatments. Exemptions include some crops such as those that are harvested before bloom (*e.g*. artichokes, lettuce), applications in enclosed spaces or insect exclusionary structures/netting, uses to control a quarantined pest, and applications under a FIFRA Section 18 emergency exemption.^82^ In 2024, the Natural Resources Defense Council (NRCD) settled litigation against the California DPR, claiming the agency had mistakenly identified neonicotinoid‐treated seed as a non‐pesticide. The settlement mandates that the California DPR review the issue and propose a plan to regulate neonicotinoid‐treated seeds by February 2, 2026, including a public comment period.^83^

#### 2.3.2 Minnesota

In Minnesota, four bills concerning neonicotinoids were signed into law in 2023. One of these bills is directly related to agriculture; HF1317/SF1339 directs state officials to develop rules and consumer guidelines for the proper use and disposal of pesticide‐treated seeds.^57^ These bills resulted from a Minnesota Department of Agriculture, Minnesota Pollution Control Agency, Minnesota Department of Natural Resources (MDA), University of Minnesota, and Board of Water and Soil Resources special registration neonicotinoid review in 2014.^84^ Ultimately, except for HF1317/SF1339, the only provisions impacting agricultural stakeholders are voluntary. Clothianidin and imidacloprid Best Management Practice guides were published in 2022, and the MDA provides a series of voluntary stewardship guidelines for soil and foliar applications and neonicotinoid‐treated seed.^84^ As of October 2024, the NRDC led the filing of a legal petition against the MDA, arguing that the MDA has failed to protect Minnesotan citizens' rights by failing to enact farther‐reaching neonicotinoid‐treated seed restrictions.^83^ However, the NRDC's petition was rejected in December 2024.^85^

#### 2.3.3 New York

In 2022, the Governor of New York signed the Birds and the Bees Protection Act (S.1856‐A/A.7640), prohibiting the use of both nitroguanidine and cyanoamidine neonicotinoids in corn, soybeans, and wheat production—a move that was anticipated to decrease the use of neonicotinoids in the state by 80–90%.^86^ Provisions to support agricultural producers were included in the bill, which does not take effect until 2029 to allow time for adaptation to its requirements.^87^ Additionally, the New York Commissioner of Agriculture may issue a waiver for the use of neonicotinoid seeds for on‐farm use, for no more than 2 years, when (1) the farm owner has completed an IPM training, (2) a pest risk assessment report has been completed, (3) neonicotinoid seeds are limited to areas identified in the pest risk assessment report, (4) the farm owner maintains records of when treated seeds are planted, (5) any applicators have taken an annual department‐approved neonicotinoid course.^87^ Exceptions also exist for environmental emergencies, including significant harm, injury, or loss to crops, and the act also requires that a study to identify practical and feasible alternatives to neonicotinoids be conducted and submitted to the state government by January 2026.^87^ The decision came after the 2020 publication of a 432‐page report by researchers at Cornell University who had been studying the issue since 2018, funded by the Environmental Research Fund.^15^

The three aims of the Cornell study were to evaluate the economic pest control and plant protection benefits of neonicotinoids in New York, estimate the risks to pollinators, and evaluate the relative economic benefits and risks of likely neonicotinoid substitutes. The study drew on 5000 paired field trials for data and found that benefits from neonicotinoids differ across crop type and application mode. For instance, they found that soil‐ and foliar‐applied neonicotinoids consistently benefited New York fruit and vegetable crops and were an important component of insecticide rotations; at the same time, neonicotinoid‐treated seeds did not consistently increase net income for New York field corn and soybean producers.^15^ Indeed, 87–93% of field trials found no increase, or in some cases even found a decrease, in yield for corn growers using neonicotinoid‐treated seeds compared to chemical alternatives or untreated controls.^15^

To assess pollinator impacts, researchers conducted a systematic literature review of over 400 peer‐reviewed studies, developed a quantitative risk assessment, and conducted new research with honeybees and bumblebees across multiple settings in New York. Findings demonstrated that neonicotinoids can, but do not always, result in risks to bees.^15^ When exposed to neonicotinoids in corn and soybean fields, 74% of these exposures negatively impacted honeybee physiology; in cucurbits and turf, honeybee reproduction was impacted in 85% and 100% of exposure cases.^15^ At the same time, different neonicotinoids present different risk levels—acetamiprid, a neonicotinoid commonly used in New York fruit crops, posed a lower risk to bees than other neonicotinoids (*e.g*. imidacloprid and thiamethoxam) and non‐neonicotinoid insecticides (*e.g*. chlorpyrifos and indoxacarb).^15^

The Cornell study found high variation among common application contexts regarding relative benefits and risks. For instance, benefits were very low in corn and soybean production while risks were relatively high; alternative pyrethroids were seen as less risky due to their non‐systemic nature.^15^ Other crop and application contexts are more balanced, while some may actually see negative environmental impacts from a neonicotinoid ban—hemlocks are cited as a significant concern.^15^ The report highlights that their intention was not to make recommendations or policy prescriptions, yet it seems clear that the findings did influence the eventual ban on neonicotinoids. Specifically, the ban parallels the lowest benefit/highest risk context identified by the study—corn, and soybean production—but does not ban neonicotinoid use in other contexts, such as cucurbits, that the study found to have a less extreme benefit/risk ratio.

Beyond the Cornell study, support for the Birds and the Bees Protection Act was broad, including religious leaders, brewers, chefs, and editorials in *The Buffalo News*, *The Times Union*, and *Newsday*.^88^ Specific supporters included the NRDC, the Sierra Club, the Northeast Organic Farming Association of New York, the National Audubon Society, the Bee Conservancy, the American Academy of Pediatrics, Mothers Out Front Capital Region, Physicians for Social Responsibility, The Climate Reality Project, and others.^89^

#### 2.3.4 Rhode Island

In 2022, the Governor of Rhode Island signed into law legislation (2022‐H 7129/2022–2299) restricting the use of both nitroguanidine and cyanoamidine neonicotinoids outdoors.^90^ The law also creates a provision allowing only certified applicators to purchase or use neonicotinoids and prohibits their use on any plant while blossoming.^90^ The law came into effect on January 1, 2024. While the National Pest Management Association opposed the bill and appeared to influence a vote delay in the House and Senate, activist groups pushed the legislature to approve it.^91^ Some concerns persist about the feasibility of enacting it, given Rhode Island's limited resources for applicator certification.^91^, ^92^

#### 2.3.5 Vermont

Although the Governor of Vermont vetoed a bill to limit nitroguanidine and cyanoamidine neonicotinoids in the state, the legislature overrode it and enacted H706, requiring that farmers get a prescription from their agronomist demonstrating that neonicotinoid‐coated seeds are necessary.^93^, ^94^ The bill explicitly prohibits the use of neonicotinoid‐treated seeds in soybeans and cereal grains, outdoor application during bloom, and outdoor application to leafy and petiole vegetable crops harvested after bloom.^95^ These provisions would go into effect in 2029 on the condition that similar legislation in New York also goes into effect and was generally modeled after New York's legislation.^96^, ^97^ H706 additionally bans outdoor applications on ornamental plants beginning in 2025. Emergency exemptions are available when particular pest threat criteria are met; specifically, any person requesting an exemption must complete an approved IPM training, complete a pest risk assessment report, plant only on land identified in said report, and maintain records of when neonicotinoid seeds are used.^95^ Exemptions cannot be valid for more than one year, must specify the type of neonicotinoid‐treated seeds allowed, and may feature additional requirements for how the seed is used.^95^ In consultation with the Agency of Natural Resources, the Agency of Agriculture, Food, and Markets may also grant an exemption if there is an insufficient supply of neonicotinoid‐free seed or if needing to purchase such seed would create undue financial hardship for farmers.^95^, ^98^

Vermont policymakers appear to have followed the model established in New York, relying on many of the same findings, including the 2020 Cornell study. The proposed Vermont regulations are similar to those in New York's Bill No. A08571, and Vermont's neonicotinoid legislation is contingent on New York's bill going into effect.^96^ The bill also cites neonicotinoid regulations in the EU, Québec, and Ontario as inspiration.^95^ There is evidence of broad public support for the bill, with a survey of Vermonters showing 83% support for a phaseout of nearly all neonicotinoids in Vermont, with exemptions available in case of emergency.^98^, ^99^

#### 2.3.6 Illinois

Illinois is another state that passed legislation that seems to be modeled on the neonicotinoid restrictions outlined by New York.^97^ The two houses of the Illinois General Assembly passed legislation in 2024 restricting corn, soybean, and wheat seed treatments that incorporate several common neonicotinoids in both the nitroguanidine and cyanomidine groups (clothianidin, imidacloprid, thiamethoxam, dinotefuran, and acetamiprid).^100^ This bill also includes provisions that allow waivers for neonicotinoid‐treated seed to be used if there is a ‘commercially insufficient amount of available seed’ lacking these neonicotinoid treatments or if this type of seed would ‘result in undue financial hardship for agricultural producers.’^100^

## 3 DISCUSSION AND CONCLUSIONS

Neonicotinoid regulations have spread from the EU to Canadian provinces and, subsequently, to specific US states, becoming increasingly voluntary and less restrictive over time. Ontario and Québec have modeled their regulations after those of the EU, and clear similarities can be found between these Canadian regulations and subsequent bills in several US states, including New York and Vermont. Notably, Vermont explicitly references Canadian regulatory frameworks.^95^ While regulatory approaches vary significantly, shared elements suggest potential trends for future policies.

There are parallels in the impetus for creating neonicotinoid regulatory schemes: concerned environmental coalitions and place‐based research. In the EU, a combination of transdisciplinary research, beekeeper and environmental group activism, journalistic attention to the plight of pollinators, and public support drove the complete ban on neonicotinoids.^21^, ^24^, ^29^, ^80^, ^87^, ^92^ European‐based public research demonstrating the negative impacts of neonicotinoids on pollinators and the insecticides' persistence in the environment were also major drivers to regulation in the EU.^24^ Similarly, Canada's regulatory schemes resulted from a growing body of Canada‐specific studies demonstrating the negative impact of neonicotinoids on bees and broad stakeholder support for regulation from public health organizations, beekeepers, farmers, and citizen groups.^50^, ^51^ In the US, a regional study conducted by Cornell University played a foundational role in shaping neonicotinoid regulatory policies in New York, Vermont and Illinois.^15^

In each case, agrichemical companies have opposed regulation, sometimes conducting private neonicotinoid‐pollinator research and orchestrating lobbying efforts. Pushback against regulations by agrichemical companies and pro‐pesticide organizations has been documented in Europe, Canada, and the US; these efforts have been credited with preventing more far‐reaching bans and instead turning regulatory approaches towards voluntary efforts and the allowance of neonicotinoid use when the need for an exemption is documented.^21^, ^28^, ^50^, ^60^, ^64^, ^83^, ^91^

Another recurring theme in agricultural neonicotinoid regulations is the use of exemptions and the legal disputes surrounding them. The EU has been particularly volatile, with exemptions shifting between allowing and prohibiting greenhouse use, permitting then banning applications in winter cereals and sugar beets, and granting individual countries the authority to issue emergency exemptions before ultimately ruling in 2023 that such exemptions are illegal.^17^, ^27^, ^28^, ^29^ Given the EU's total ban on nitroguanidine neonicotinoids, the fight over exemptions is unsurprising, as stakeholders seek any available loophole.

Canadian provinces and US states have typically incorporated exemptions into their neonicotinoid regulations, but legal disputes persist in some areas. Ontario, Québec, California, New York, Rhode Island, Illinois, and Vermont all have regulations that allow the use of some neonicotinoids under various conditions as a matter of course, including agronomist‐verified need, pest risk assessment reports, applicator/farming education and training, record keeping, and emergency situations such as particular pest threat thresholds or undue economic strain on farmers.^52^, ^54^, ^82^, ^87^, ^90^, ^96^ In some cases, such as Minnesota, bans only apply to foliar and soil treatments but do not address seed treatments.^84^ The NRDC has filed legal petitions addressing such exemptions in California and Minnesota. At the federal level, the Pesticide Action Network has pushed the EPA more broadly on neonicotinoid regulation.^33^ Legal battles, it seems, are destined to be a central feature of any attempts to regulate the use of neonicotinoids.

Many laws enforcing neonicotinoid bans and restrictions have been enacted so recently that assessing their effectiveness in reducing neonicotinoid use and their agronomic, economic, environmental, and health impacts remains complex. Additionally, existing research suggests that these impacts are highly context‐dependent, varying based on cropping systems, climate, and the availability of alternative pest management options. For example, while studies indicate that neonicotinoid bans have significantly affected canola yield and profitability in the EU, Sweden defies this trend—showing no discernible impact on cropping area or yields, possibly due to its cooler climate and limited insect resistance to pyrethroids.^39^, ^40^, ^42^ Similar findings demonstrating no discernible impact of neonicotinoid restrictions on cropping area or yields have been reported in Québec.^53^, ^58^

The replacement of neonicotinoids and the associated changes in toxicity risk have also varied across regions. After restricting neonicotinoids, many countries have shifted to pyrethroids or diamides, sometimes at higher usage levels than neonicotinoids, raising concerns about increased pollinator toxicity risk.^43^, ^44^, ^45^, ^46^, ^47^, ^48^, ^59^ However, Germany's overall pollinator toxicity risk has declined following the neonicotinoid ban.^49^ In response to the shift toward diamides, Québec has announced plans to require a prescription from a trained agronomist for all insecticidal seed coatings starting in 2025.^53^ Currently, no US data exist on the impacts of neonicotinoid regulations, but Grout *et al*.'s Cornell study demonstrates that environmental, agronomic, and economic outcomes can vary widely by commodity.^15^

What can we infer from these trends? For one, the nature of regulation will largely depend on each government's adherence to the precautionary principle. The EU has taken a highly precautionary approach by instituting a complete ban, while Canadian and US federal agencies have been slow or reluctant to act, leaving regulation mostly to lower‐level regional governments. In this regulatory gap, some private national and international corporations have implemented their own pollinator protection policies.^71^, ^73^, ^74^ Overall, most Canadian provinces and US states have accepted potential risks in exchange for the benefits of agricultural neonicotinoids; currently, only two of 10 provinces and five of 50 states have laws regulating their agricultural use. Even where such regulations exist, exemptions for needs‐based scenarios are typically written into the law. Secondly, similar drivers have spurred the development of neonicotinoid regulation. In the EU, Canada, and the US, neonicotinoid regulations have typically been compelled by a combination of factors: (1) the availability of context‐specific impact studies, (2) lobbying efforts by environmentalist and agrichemical industry groups, (3) public support, and (4) the regulatory approaches of other regions. These factors may make regional governments more likely to adopt a precautionary stance on the use of neonicotinoids.

More time and research are needed to determine where future neonicotinoid bans will emerge and what their consequences will be. This overview is intended as an initial step toward understanding these developments. As consumer awareness, media coverage, and corporate interest in neonicotinoids increase, it is likely that regulatory proposals will continue to evolve, drawing upon and diverging from the examples discussed in this review.

## CONFLICT OF INTEREST

The authors declare no conflicts of interest

## Data Availability

Data sharing not applicable to this article as no datasets were generated or analysed during the current study.
